# Fifty years of struggle to control cutaneous leishmaniasis in the highest endemic county in Iran: A longitudinal observation inferred with interrupted time series model

**DOI:** 10.1371/journal.pntd.0010271

**Published:** 2022-04-29

**Authors:** Mohammadreza Aflatoonian, Iraj Sharifi, Behnaz Aflatoonian, Ehsan Salarkia, Ahmad Khosravi, Razieh Tavakoli Oliaee, Mehdi Bamorovat, Abbas Aghaei Afshar, Zahra Babaei, Fatemeh Sharifi, Moslem Taheri Soodejani, Mohammad Reza Shirzadi, Mohammad Mehdi Gouya, Abolhassan Nadim, Hamid Sharifi

**Affiliations:** 1 Leishmaniasis Research Center, Kerman University of Medical Sciences, Kerman, Iran; 2 Research Center for Tropical and Infectious Diseases, Kerman University of Medical Sciences, Kerman, Iran; 3 Center for Healthcare Data Modeling, Departments of Biostatistics and Epidemiology, School of Public Health, Shahid Sadoughi University of Medical Sciences, Yazd, Iran; 4 Center for Diseases Control, Ministry of Health and Medical Education, Tehran, Iran; 5 Department of Epidemiology and Biostatics, School of Public Health, Tehran University of Medical Sciences, Tehran, Iran; 6 HIV/STI Surveillance Research Center, and WHO Collaborating Center for HIV Surveillance, Institute for Futures Studies in Health, Kerman University of Medical Sciences, Kerman, Iran; Charité University Medicine Berlin, GERMANY

## Abstract

Negligible data are available following major social activities and environmental changes on leishmaniasis. Therefore, how interactions between these events influence cutaneous leishmaniasis (CL) risk is not well-known. This longitudinal study was undertaken to explore the impact of interventions conducted between 1971 and 2020 in Bam county, which has had the highest disease burden in Iran. Only confirmed CL cases during this period were taken into account. Data were analyzed by SPSS 22 using the X^2^ test to assess the significance of the difference between proportions. Moreover, we used interrupted time series (ITS) to assess the impact of three environmental events during this period. Overall, 40,164 cases of CL occurred in the past five decades. Multiple complex factors were among the leading causes that synergistically induced the emergence/re-emergence of CL outbreaks in Bam. The main factors attributed negatively to CL control were cessation of malaria spraying activity, expansion of the city spaces, and a massive earthquake creating new breeding potentials for the vectors. The highest impact on CL incidence during these years was related to the earthquake [coefficient = 17.8 (95% CI: 11.3, 22.7); p-value < 0.001]. Many factors can contribute to CL outbreaks in endemic foci. They also can cause new foci in new areas. Since humans are the single reservoir for CL in this area, early detection and effective management significantly contribute to controlling CL to reduce the disease burden. However, essential evidence gaps remain, and new tools are crucial before the disease can ultimately be controlled. Nevertheless, sustained funding and more trained task forces are essential to strengthen surveillance and case management and monitor the interventions’ impact.

## Author summary

We aimed to conduct this longitudinal observation to assess the impact of interventions applied between 1971 and 2020 in Bam county, Iran’s highest anthroponotic cutaneous leishmaniasis burden. Only confirmed CL cases were taken into account. Overall, 40,164 cases of CL occurred in the past five decades. Multiple complex factors were among the leading causes that synergistically induced the emergence/re-emergence of CL outbreaks in Bam. The main factors attributed negatively to CL control were cessation of malaria spraying activity, expansion of the city spaces, and a massive earthquake creating new breeding potentials for the vectors. Since humans are the single reservoir host for CL in this area, early detection and effective management significantly contribute to controlling CL to reduce the disease burden. However, continuous funding and more trained forces are critical to strengthening surveillance and case management and monitoring the interventions’ impact.

## Introduction

Neglected tropical diseases (NTDs), including leishmaniasis, are closely linked with human behavior, urbanization, and environmental changes, both man-made (anthropogenic) and natural disasters [1,2]. Increasing determinant factors are associated with multiple and complex modifications that together make leishmaniasis a leading social and severe public health concern in more than 101 tropical and subtropical countries and territories with one billion affected people [3,4]. Various clinical forms of leishmaniasis depend on the causative parasite, vector species, and ecological niche. Among them, visceral form (VL) and cutaneous leishmaniasis (CL) are globally important diseases [4].

Cutaneous leishmaniasis is the most widely distributed type, and the majority of cases occur in West and Southeast Asia, the Americas, and East and North Africa [5]. CL exists in two fundamental types in the Old World: zoonotic (ZCL) produced by *L*. *major* and found principally in small rodents as the primary reservoir host. In contrast, anthroponotic CL (ACL) is found predominantly in humans as the primary host transmitted by the female *Phlebotomus sergenti* sandfly in an anthroponotic life span [6]. The latter is essentially an urban and peri-urban form, found in large and medium-sized cities, representing spatial clustering patterns in Southeast Asia. The disease is common in fragile health systems and presents self-healing lesions, life-long stigmatization, distress, and permanent skin alterations [7]. Large outbreaks characterize the disease in heavily populated municipalities, particularly battle zones, peri-urban settlements, and disaster-affected people [8,9].

In developing countries, mass human migration flows have recently led to unexpected expansion challenges of heterogeneous ’megacities,’ where living standards for housing and sanitary conditions are insufficient. Therefore, such environments create suitable breeding conditions, and enhance human exposure to transmitting vector-borne diseases (VBDs), including leishmaniasis [10,11]. In addition, urbanized areas and disasters promote increased movement of people, often animals and wildlife products, and relevant diseases between rural and urban locations. They are responsible for widespread land degradation, agricultural failures, water shortages, livestock loss, and enhanced outbreaks of diseases [12].

Unfortunately, rudimentary data indicate the health consequences of disaster events or the real impact of ecosystem instability and other risk-related disasters. However, many confounding factors, such as trade and travel, civil unrest, migration, low socioeconomic status, unplanned growth, ecological alterations, and climate changes, are significant drivers and force people to leave their original homelands and abandon their villages [13,14]. This phenomenon is a social and biological process that takes place over time through greater concentrations and connectedness of humans and alters all society sectors. In such affected populations, migration patterns evolve from being frequently rural-to-urban or urban-to-urban and often to the shantytowns and peri-urban neighborhoods with little or no access to civil services. [15].

Numerous other risk factors play a pivotal role in intensifying leishmaniasis in ecologically sensitive areas within endemic countries. These causes include insufficient reservoir and vector control, natural disasters, population displacement, lack of sanitary conditions, solid waste management, social and behavioral hazards, and resistance to conventional drugs. Natural disasters, tsunamis, earthquakes, droughts, floods, and cyclones can produce appropriate environments for amplifying infectious diseases and result in epidemics [8,14–21]. Such changes contribute to favoring the epidemic, spread, and the emergence of CL.

Iran is among the most disaster-emergent countries to earthquakes, droughts, and wind storms [16–22]. On the other hand, CL is the most epidemic-prone and poverty-stricken VBD [23,24]. Hence, these combinations readily provide multiple biological and ecological confounding factors regarding the CL emerging outbreaks [25]. In addition, these emerging outbreaks show the evolutionary potential and dynamic interactions between the *Leishmania* parasite, its host, and the environment. For example, the majority of identified CL cases have been *Leishmania tropica* causing ACL in Bam [17–22,26].

The present study aimed to explore a long-term historical observation (1971–2020) of CL evolution in a well-known focus in Bam and show how human behaviors and urbanizations combined with disasters synergistically induced epidemic conditions and facilitated preventive and therapeutic measures to control the disease. Lessons learned through this study and the implementation of robust interventions could be a suitable model for designing control strategies against CL, notably in the Eastern Mediterranean Region (EMR), where approximately 80% of the overall case counts are reported [5].

## Material and methods

### Ethical statement

The Kerman University of Medical Sciences’ Institutional Review Board permitted this study (Ethics no. IR.KMU.REC.1400.035). All the CL patients were regularly visited, diagnosed, and treated free of charge with proper drugs accordingly. Patients with other problems were referred to the higher health clinics or hospital levels for further diagnosis and treatment regimens. Furthermore, parents/guardians gave verbal informed consent "on behalf" of all the children who took part. The cases that agreed to participate were recorded.

### Study area

**Fig 1:**
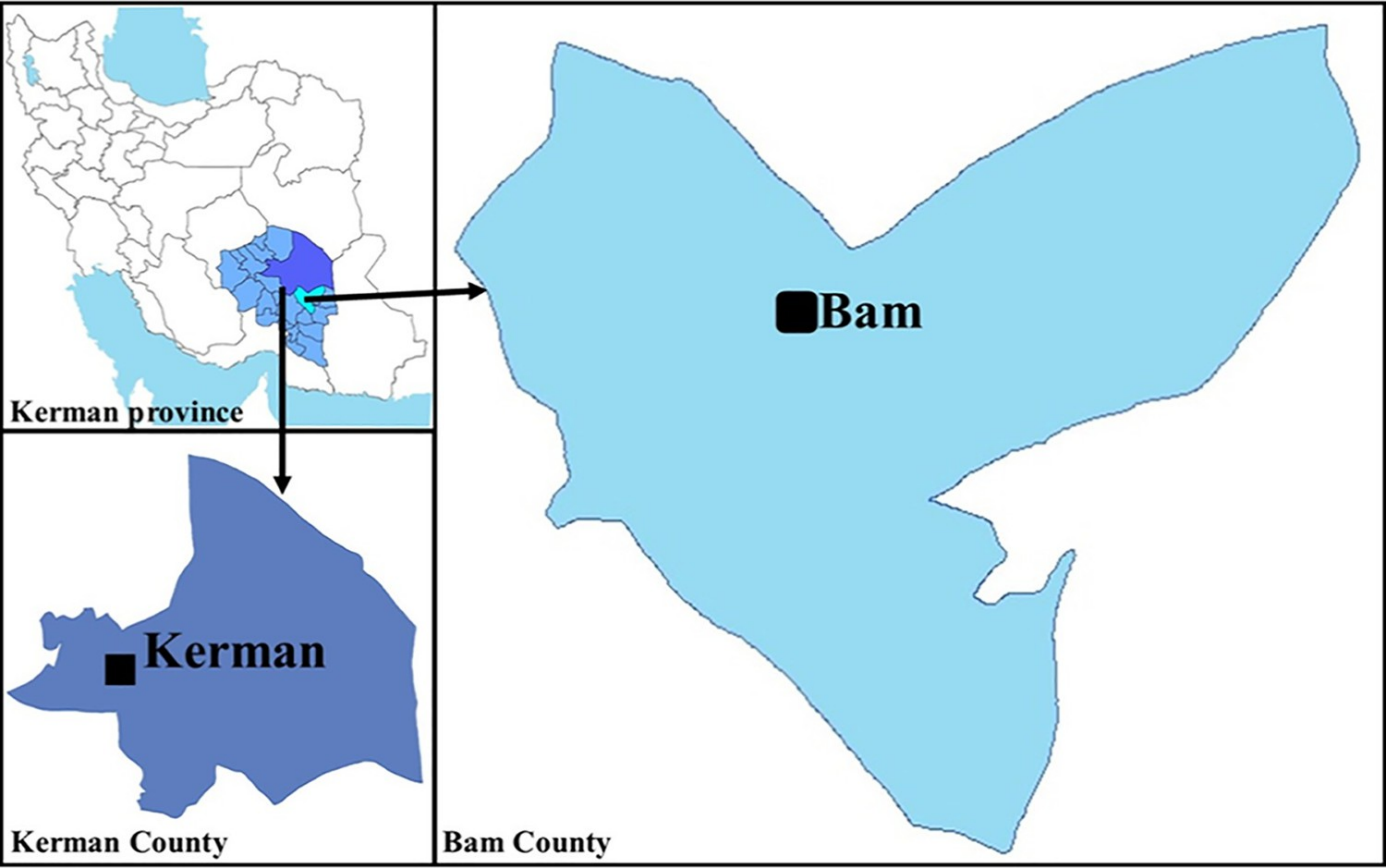
The affected areas (◼) within Bam county and peri-urban areas in Kerman city, southeastern Iran. GIS layers prepared by the National Cartographic Center of Iran (https://www.ncc.gov.ir/).

**Fig 2:**
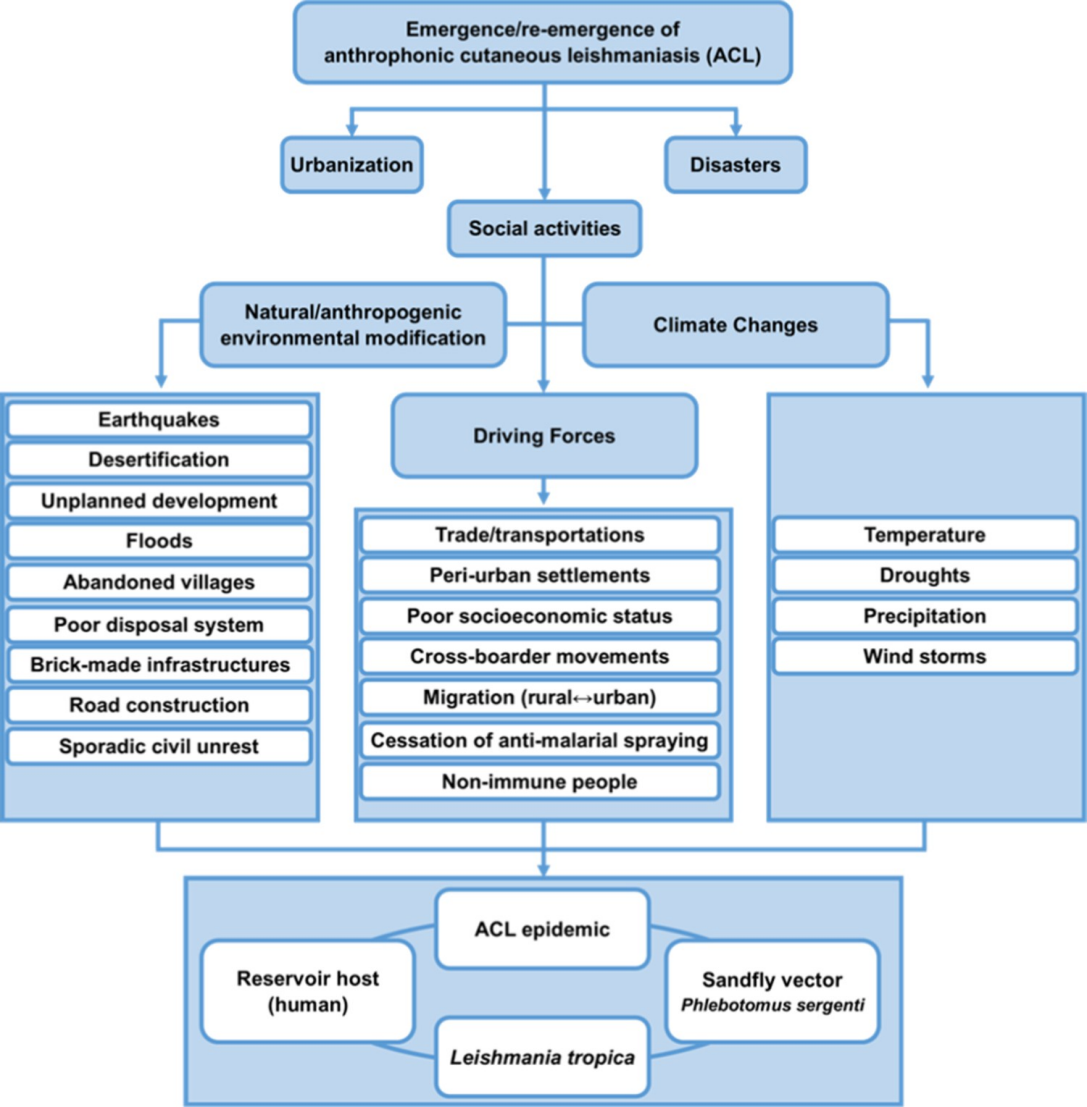
Multiple environmental alterations and social activities were major forces for the induction of emergence/re-emergence of anthroponotic cutaneous leishmaniasis repeated outbreak waves in Bam, southeastern Iran.

**Fig 3:**
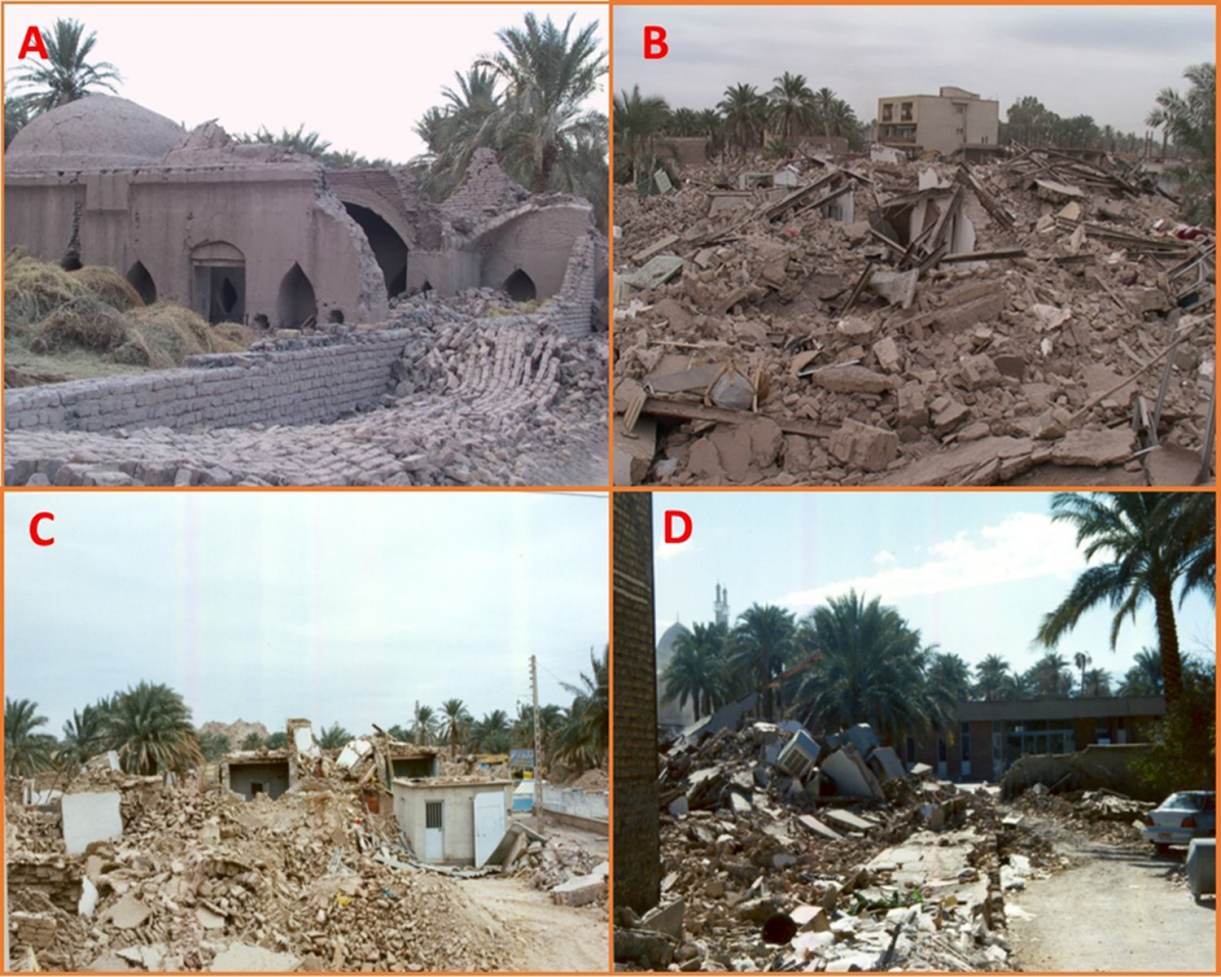
Images of the earthquake destroyed 90% of raw brick-made houses and created a suitable sandfly breeding condition for transmission of anthroponotic cutaneous leishmaniasis in Bam, southeastern Iran (the research team took the above pictures in 2003 following the earthquake of Bam).

This investigation was undertaken in Bam county (Fig 1), 1,200 km from Tehran, southeastern Iran [27]. Several environmental and social activities have occurred in the area (Fig 2). Subsequently, three major remittent outbreak relapses have occurred in Bam county, more significantly due to cessation of malaria spraying action, urbanization, the large-scale plan to widen the city spaces, the massive earthquake, and ongoing droughts. The basic structure of the city of Bam is built of raw brick (Fig 3), including the Citadel Arc-e-Bam. Cutaneous leishmaniasis history goes back to nearly 100 years ago when people used to live in the Citadel Arc-e-Bam, which is the largest mud-brick complex in the world, with a massive fort, at the heart of which the central Arc (castle) is located as the highest sector; however, the name "Arc-e Bam" refers to the whole construction.

The earthquake in Bam damaged approximately 90% of the civil infrastructures and natural resources such as clinical and health facilities, and water supply and disrupted the solid waste management disposal system in 2003 [28]. The economic loss of the earthquake was estimated at around 5% of the country’s gross national product (GNP). Such losses aggravated underdevelopment and triggered a mass migration from rural to urban areas and vice-versa within the Kerman province. Simultaneously, 65,000 people, 35,000 from rural areas and 30,000 mostly from different non-immune provinces, arrived in the city for various reasons.

### Historical background and data sources

This population-based longitudinal observation was conducted from June 1971 to December 2020 for 50 years in Bam county. Initially, a CL health unit was established in 1993 for diagnosis and treatment purposes in Bam. Then in 2005, it was organized into a CL Health Clinic in charge of all activities. This clinic has been linked with the Faculty of Medicine, Kerman and Bam Universities of Medical Sciences, and the Leishmaniasis Research Center. The clinic has been the central registry system that accomplishes CL cases referred from diverse urban and rural communities. Data were obtained from the registry of Bam Health System. All CL cases have been confirmed by direct microscopic smear preparation.

Before 1993 health records were traditionally written on paper and maintained in folders for database *backup*. However, after 1993, the accuracy and quality of data recorded significantly improved after establishing a digital registry system.

### Integrated vector control

Leishmaniasis is a notifiable disease in Iran and has firmly been integrated with PHC services in Bam. The vector control strategy aims to reduce or interrupt the spread of the parasite in domestic and peridomestic transmission habitats. A combination of control measures has been employed in high-risk localities as follows:

### Personal protection

Diethyl-meta-toluamide (DEET) has been given to the CL patients at the clinic. They were advised to use insecticide-impregnated bed nets, door fences, windows, curtains, and clothing (61). The county health system distributed bed nets among the inhabitants in high-risk localities within the city of Bam. Additionally, they prepared themselves their nets. The impact of the personal protective measure was evaluated sporadically. One of the most prophylactic training methods in the health care system has been face-to-face education by the health staff. The majority of people in the affected areas can be familiar with the disease, the preventive and therapeutic measures. The staff has had to do face-to-face learning at least once every year for the member of households in the high-risk areas.

### Public awareness

In the affected areas of Bam, information has been disseminated by PHC workers as a regular task through different means such as social media (local newsletters, television, and radio), leaflets, posters, brochures, and campaigns. Also, health volunteer forces who are residents and trained with professional skills participated actively in health-related public issues. As coordinated by the higher health authorities and community engagement, many activities have been carried out, notably public awareness and advocacy.

### Chemicals and environmental management

Before 1970, insecticide spraying was employed before to transmission season against malaria and leishmaniasis vectors simultaneously two times a year. The first round of spraying was done in April and the second in September. Dichlorodiphenyltrichloroethane (DDT) was the leading insecticide applied at 2 grams per square meter (m^2^). Due to malaria elimination in Bam county, long-term spraying of domestic and peridomestic dwellings was terminated. However, environmental health personnel has been responsible for selective indoor residual spraying (IRS) of houses with pyrethroids (permethrin) sustained when referred patients with CL have been confirmed infected. Due to severe infestations of date palms with Dubas bugs (*Ommatissus* spp.) in Bam, permethrin and other pyrethroids have extensively been used as above at similar doses and times to spray palm trees in the county systematically.

The removal of garbage left around the houses, solid wastes, debris, cleaning the streets/passages, and dog population management have been the responsibility of the municipal department.

### Case detection and management

In this study, the causative species has ever been *L*. *tropica*. For this reason, the health surveillance system has established a model and an efficient functioning control clinic for early detection, reliable diagnosis, free of charge treatment of identified cases, and six months follow-up of treatment outcomes.

### Treatment of patients

The therapy of cases was carried out either by daily administration of 20 mg/kg of meglumine antimoniate (MA) for 21–28 days intramuscularly or by intralesional administration of MA every week for 8–12 weeks along with liquid nitrogen cryotherapy biweekly, depending on the location, size, and the number of cutaneous lesions [29]. A combination of other drugs has been assigned for patients with underlying complications or non-healing CL forms.

### Epidemic preparedness and response

Periodic epidemics of ACL induced by *L*. *tropica* have occurred in the area and adjacent counties [17–22]. The occurrence of an outbreak is problematic to forecast. The events involved include variations in vector habitation, mass relocations of people due to drought, cross-border movements from neighboring endemic countries such as Afghanistan and Pakistan, intra-migration, and poor immunity. Therefore, it is essential to emphasize establishing tools to forecast outbreaks, identify them at early commencement, promptly respond to outbreak situations, and prevent any abnormal situations related to the disease. Basic preparedness and rapid response appliances have been in place since 1993 in Bam county, detecting CL patients timely and reacting rapidly to emergencies. In vulnerable areas of Bam, the responsibilities of outbreak task forces, the necessary needs for a response, surveillance, and control measures, and all health facilities have relatively been delivered with the least possible diagnostic tools and treatment materials.

### Basic and operational research

Widespread research efforts have been made in the affected area, with assistance from Kerman and Bam Universities of Medical Sciences, Health Services, and research institutes, to detect risks, the ACL burden, and the vulnerable population. Particular important aspects include an assessment of risk factors, studies of the local burden, epidemiological data, vectors incrimination, *Leishmania* species identification from primary hosts (humans) in new emerging foci of CL, operational research to estimate drugs and treatment schedules, approaches for sandfly and reservoir control and surveillance/monitoring tools, socioeconomic and behavioral research on the use of healthcare facilities and research addressing efficacy of conventional and novel control strategies [30–34].

### Miscellaneous activities

Other extensive struggles to manage ACL in Bam have fairly been made to control the disease in the past decades to reduce the disease burden, including municipal participation, capacity-building, health personnel training, public education, cross-border collaboration, intersectoral partnership, organization action, and monitoring/evaluation.

### Statistical analysis

Data were analyzed by SPSS 22 (Chicago, IL, USA) using the X^2^ test to assess the statistical significance between proportions. Possible anthropometric and disease features for patients with CL were evaluated. In addition, the new cases (incidence) with CL among different age ranges, sexes, numbers, and sites of lesions were assessed. *P*-value < 0.05 was defined as statistically significant. We used an interrupted time series (ITS) model, using R software (version 4.1.1) (http://www.aimjournal.ir/Article/aim-18671), for multiple interventions to assess the impact of three events (cessation of anti-malaria spraying in 1977, widening of the spaces, 1990–1991, and earthquake in 2003) in Bam county during 1971–2020. We entered all three events in the model and assessed the relevant impact on the trend of the CL incidence. In addition, we fitted several models to assess the best lag between the events and the change in CL incidence. The models showed that the best-fitted model has a two-year lag after the events.

In this model, *β*_0_ shows the intercept or incidence of leishmaniasis in 1971. *β*_1_ represents the trend of leishmaniasis incidence without consideration of these three events. *β*_2_ denotes the level change after two years pre- vs. post-cessation of anti-malaria spraying in the region. *β*_3_ displays the change in the CL trend after the cessation of anti-malaria spraying. *β*_4_ presents the level of change after two years pre vs. post a large-scale widening of the city space. *β*_5_ performs the changes in the CL trend after a large-scale widening of the city space. *β*_6_ displays the level of change after two years pre- vs. post-earthquake. *β*_7_ exhibits the trend after the earthquake, and ε is a random error term of the model.

$${Outcome=\beta }_{0}+\beta_{1}(time)+\beta_{2}(level1)+\beta_{3}(level1*time)+\beta_{4}(level2)+\beta_{5}(level2*time)+\beta_{6}(level3)+\beta_{7}(level3*time)+\varepsilon .$$ Formula 1

## Results

Overall, 40,164 cases of ACL occurred in the past five decades, between 1971 and 2020, in Bam, southeastern Iran. The average annual cases were approximately 893 in a median population of 93,000 (Table 1). Three major man-made and natural events occurred in the past half-century; cessation of anti-malaria spraying activities (the first outbreak), execution of large-scale broadening of the streets and passages within Bam city, and a massive earthquake in the area that induced several outbreaks of cases significantly (p < 0.001).

**Table 1:** Representing the baseline population of patients with cutaneous leishmaniasis in Bam, southeastern Iran, 1971–2020.

Period (year)	Median population	Period (year)	Event	Annual incidence (per 1,000)
**1971–1979**	60,500	9	Low endemic	3.31
**1980–1983**	63,200	4	1^st^ outbreak	12.51
**1984–1989**	75,800	6	Control period	9.14
**1990–1993**	78,800	4	2^nd^ outbreak	22.75
**1994–2004**	98,600	11	Control period	7.68
**2005–2008**	102,500	4	3^rd^ Outbreak	20.52
**2009–2020**	125,000	12	Emergence of new foci	4.75
**Average**	93,000	50	---	9.60

All age groups, genders, number of lesions, and anatomical sites of lesions were equally affected with a similar incidence pattern in patients with ACL in the endemic period (before outbreaks) and following the termination of spraying activities against malaria and expanding the city spaces. In contrast, the incidence of new cases demonstrated a significant tendency towards higher age groups (p < 0.001), males (p < 0.001), and hands (p < 0.001) after the earthquake. However, no change was found when the number of lesions was considered. Most lesions were single, followed by two and ≥ 3 lesions (Table 2). Thus, demographic and clinical characteristics in endemic periods and after the first two outbreaks were generally similar to the typical feature of the disease in the control period.

**Table 2:** Representing age groups, genders, the number of lesions, and anatomical location of lesions in cases with anthroponotic cutaneous leishmaniasis in Bam, southeastern Iran.

Characteristics/Events		Endemic status (%)	Cessation of anti-malaria spraying (%)	A plan of widening streets (%)	Earthquake (%)	P-value*
Age	<6	15	15	11	7	< 0.001**
	6–20	36	34	43	28	
	≥21	49	51	46	65	
Gender	Female	50	55	58	42	< 0.001**
	Male	50	45	42	58	
No of lesions	1	75	78	75	60	0.19
	2	17	15	17	25	
	≥3	8	7	8	15	
Anatomical sites	Face	62	47	49	28	< 0.001**
	Hands	28	42	38	59	
	Legs	8	10	11	11	
	Other	2	1	2	2	

*Comparisons were made using a chi^2^-test.

**The p-value of all two-by-two comparisons was <0.001.

**Fig 4:**
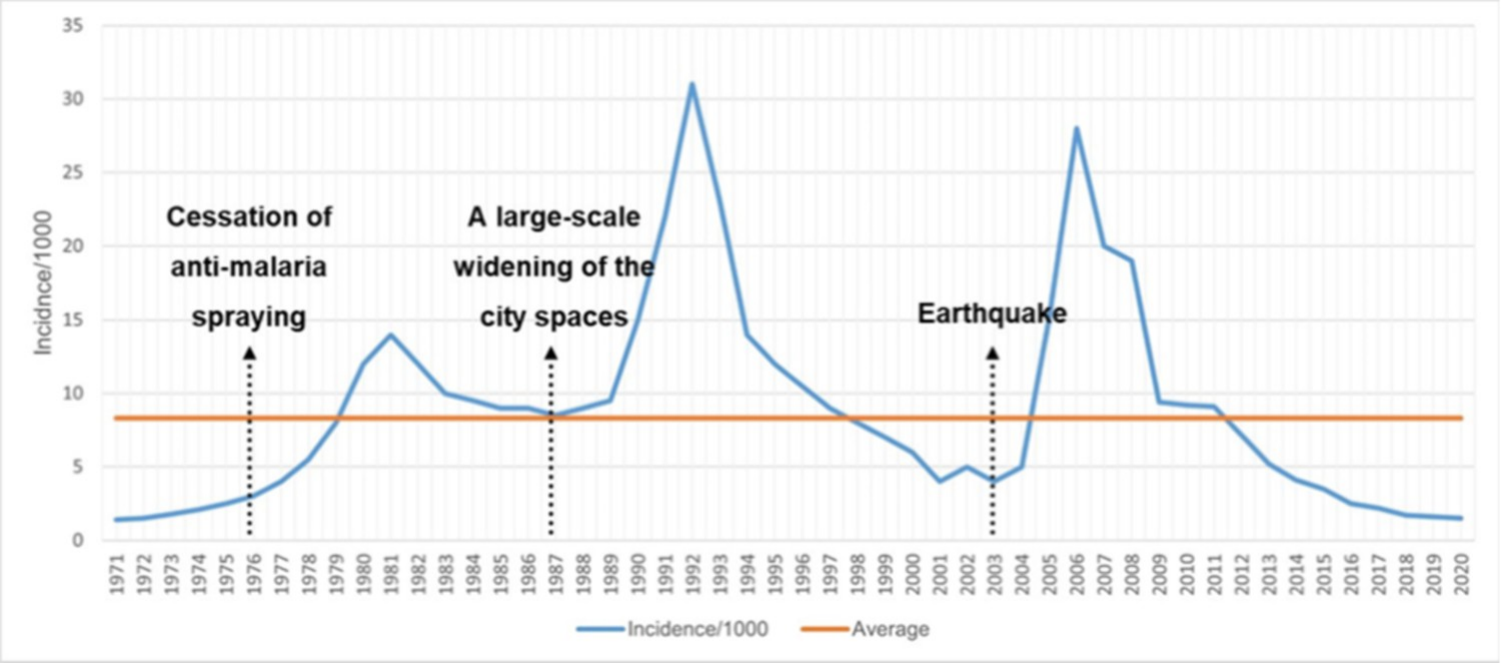
Repeated outbreak waves following the cessation of spraying against malaria, an extensive widening of the city spaces, and an earthquake occurred in Bam, southeastern Iran (1971–2020).

During the study period, three distinct and drastic rises of the cases occurred in the area; the first outbreak with slow onset commenced sometime in 1979, closely associated with halting the malaria spraying venture and reached the peak in 1981 and declined rather slowly after that. On the other hand, the second vast outbreak initiated abruptly following the broadening of the municipal spaces, particularly the streets of the city in 1988, outreached its peak in 1992 and sharply decreased the average case counts (Fig 4). Finally, the last outbreak occurred one year after the massive earthquake in 2004 and reached the maximum peak in 2006, and suddenly declined to the meso-endemic circumstance where extensive and vigorous preventive/therapeutic activities were implemented and sustained.

**Fig 5:**
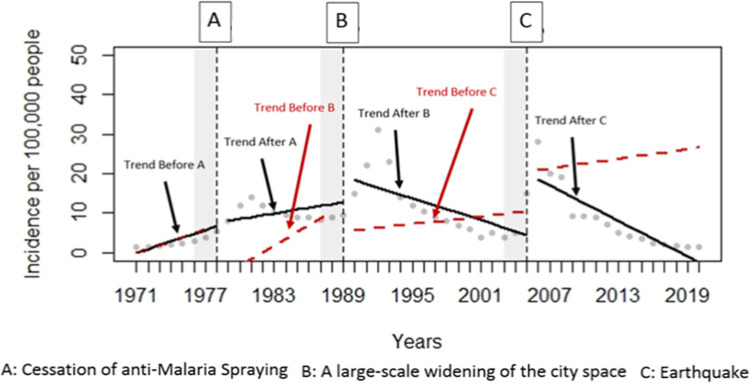
Incidence of cutaneous leishmaniasis (CL) cases before and after three major events. Vertical lines indicate the two years after every event; dotted line indicates the observed incidence of CL; dashed line indicates the trend before every event; solid line indicates the trend after every event in Bam, southeastern Iran (1971–2020).

The incidence of leishmaniasis during 1971 to 2020 had some increasing or decreasing patterns. Two years after the cessation of anti-malaria spraying, there was an increasing pattern in the incidence of the disease [coefficient = 7 (95% CI: 0.0, 14.1); p-value = 0.05]. The trend after this event had a decreasing pattern but it was not statistically significant [coefficient = -0.8 (95% CI: -2.2, 0.6); p-value = 0.28]. The large-scale widening of the city space had more impact than the previous event. The incidence of the disease had an increasing pattern after two years of the event [coefficient = 13 (95% CI: 6.9, 19.1); p-value < 0.001]. The disease had a decreasing pattern after the second event. This reduction in the incidence was statistically significant [coefficient = -0.9 (95% CI: -1.8, -0.1); p-value = 0.04)]. The highest impact on leishmaniasis incidence during these years was related to the earthquake’s third event. The incidence of CL had a considerable increase after the earthquake [coefficient = 17.8 (95% CI: 11.3, 22.7); p-value < 0.001]. Although the incidence of new cases displayed a substantial decreasing pattern after the earthquake but it was not statistically significant [coefficient = -0.42 (95% CI: -1.1, 0.2); p-value = 0.19)] (Tables 3 and 4 and Fig 5).

**Table 3:** Interrupted time series parameters to evaluate trends in incidence per 1,000 of cutaneous leishmaniasis in Bam, southeastern Iran (1971–2020).

Variable	Coefficient Intervals (95% CI*)	P-value
Intercept (***β***_**0**_)	0.3 (-5.8, 6.4)	0.9
Trend before cessation of anti-malaria spraying (***β***_**1**_)	0.54(-0.7, 1.7)	0.38
Level change after 2 years pre- vs. post-cessation of anti-malaria spraying (***β***_**2**_)	7(0.0, 14.1)	0.05
Change in trend after cessation of anti-malaria spraying (***β***_**3**_)	-0.8(-2.2, 0.6)	0.28
Level change after 2 years pre vs. post a large-scale widening of the city space (***β***_**4**_)	13(6.9, 19.1)	<0.001
Change in trend after a large-scale widening of the city space (***β***_**5**_)	-0.9(-1.8, -0.1)	0.04
Level change after 2 years pre- vs. post-earthquake (***β***_**6**_)	17.8(11.3, 22.7)	<0.001
Change in trend after earthquake (***β***_**7**_)	-0.42(-1.1, 0.2)	0.19

*95% Confidence intervals.

**Table 4:** Changes in cutaneous leishmaniasis (CL) incidence per 1,000 after three major events in Bam, southeastern Iran (1971–2020).

Time	Changes in Incidence of CL (95% CI*)		
	cessation of anti-malaria spraying	anti-malaria spraying	Earthquake
End of 2^nd^ Years	6.2 (0–18.2)	13.1 (0–27)	17.4 (0–24.9)
End of 3^rd^ Years	5.4 (0–17.4)	14.1(0.3–27.8)	17 (0–23.5)
End of 4^th^ Years	4.6 (0–16.6)	15.1(1–28.8)	16.5 (0–22)
End of 5^th^ Years	3.8 (0–15.4)	16.1 (2–29.8)	16.1 (0–23.1)
End of 6^th^ Years	3 (0–14.9)	17.1 (3–308)	15.7 (0–21.9)

*95% Confidence intervals.

**Fig 6:**
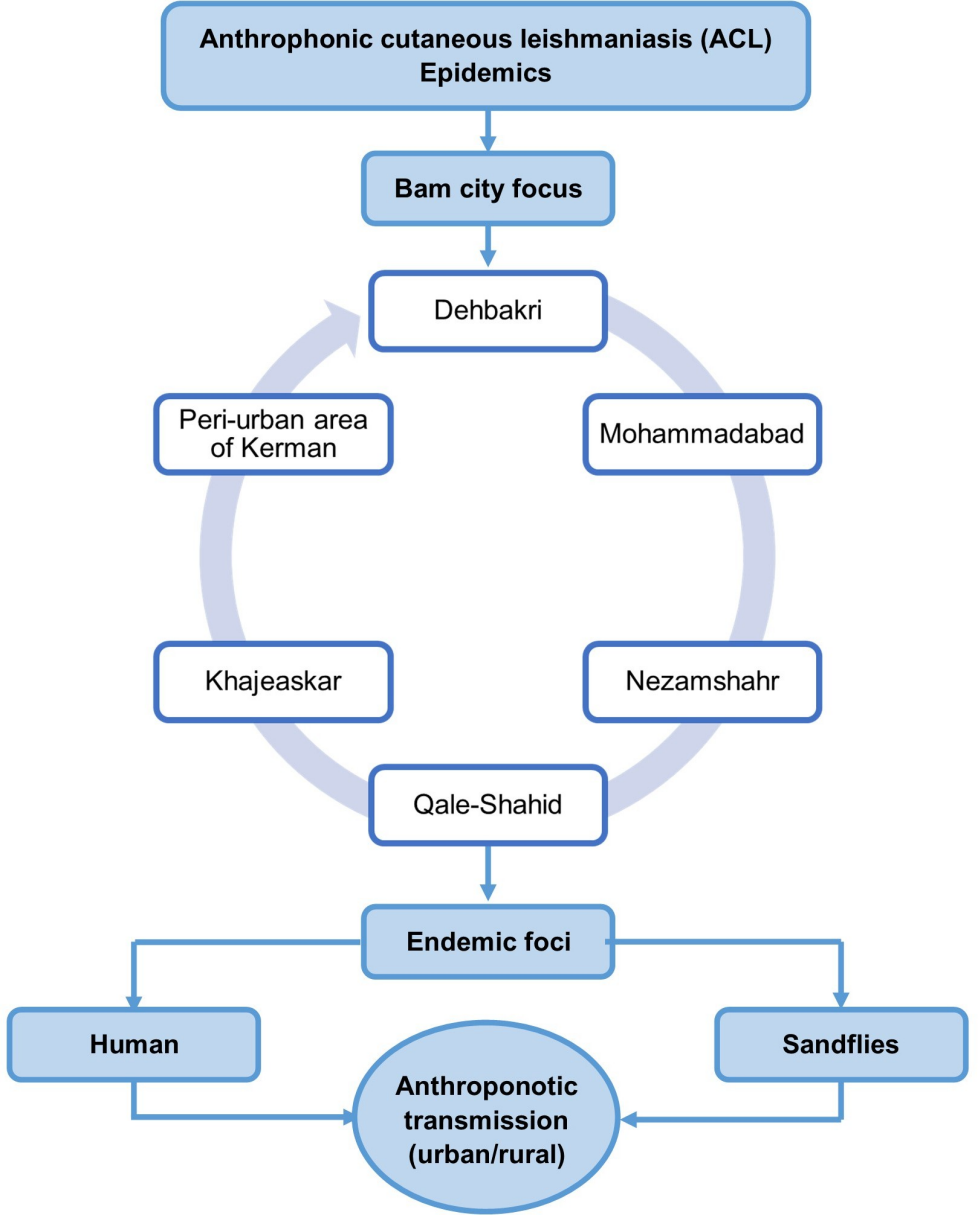
Illustrating the sequence of emerging foci in order of occurrence in Bam, southeastern Iran (1971–2020).

The spread and emergence of cases in towns and peri-urban areas within the Bam county and Kerman province correlated with the movement of people from rural to urban and conversely from urban to rural communities where they constituted new foci (Fig 6). Anthroponotic CL involves different age ranges and all year-round. Most of the skin lesions were typically localized (95%) and evolved 8–10 months; however, to a minor extent, lupoid leishmaniasis (4–5%) lasted for many years, and the remaining other atypical forms (0.5%).

## Discussion

The results indicate that CL has been endemic in Bam county for a half-century. However, many precipitating factors were created following the cessation of anti-malarial spraying, an extensive widening of the city spaces, and an earthquake that triggered repeated outbreaks of CL in Bam, southeastern Iran. Leishmaniasis is a mysterious disease associated with numerous risk factors [35,36]. The spatial distribution of CL is unique as reported to emerge/re-emerge and often expands its traditional endemic boundaries, particularly in the southern parts of Iran [37]. The past decades have witnessed extensive changes in human beings’ lives, and rapid growth has occurred in most countries. Unplanned urbanization, the incidence of significant events, limited access to health services, landscape degradation, and other trends created many outbreaks of infectious agents. One of the most outstanding topographies of the last few decades has been the fast urbanization rate in this area and abroad. The pattern of urbanization and socioeconomic conditions can accelerate disease transmission-concentration of humans, contributing to inequity in distributing clinical and healthcare services and promoting hotspots of diseases within cities for the speedy spread of emerging infectious diseases [10,12].

Scarce data are available following significant disasters or ecological factors. Therefore, how interactions between environmental and socioeconomic issues impact CL risk is unknown. Following the events, sandfly adults find appropriate resting and breeding sites in domestic and peridomestic dwellings where predisposing factors become available [38,39]. Due to carbon dioxide emissions, growing population masses in outlying neighborhoods, especially in densely populated areas, could attract phlebotomine species [40]. Moreover, outskirts settlements under deprived local hygienic conditions, poor living standards, and inappropriate housing environments could increase sandfly resting and breeding places and enable the transmission of the disease to humans. Urbanization contributes to tremendous challenges in the epidemiology of communicable diseases [12]. New so-called megacities potentially become mega-incubators for the emergence of new outbreaks and, in turn, to spreading zoonotic diseases faster. Global investigators have called the trend "an emerging humanitarian disaster" [10]. The significant drivers are complicated social-environmental disturbances and disaster events [10,41–45].

The urban area of the city of Bam has dramatically expanded. This exponential growth has a remarkable impact on local health; urban areas have become important hubs for transmitting ACL because of migration and travel. Iran is among the deadliest and most disaster-prone countries with 27,000 alone in the 2003 earthquake in Bam [46]. The earthquake was graded as the most horrible documented disaster in recent Iranian history, a catastrophic fact in a country already categorized as the world’s 4^th^ most disaster-prone nation [47]. Many of the disaster-affected population from Bam county left the area and resided in peri-urban areas adjacent to Kerman, where ACL was endemic. As a result, a long-lasting epidemic of ACL emerged [8].

There is evidence to ensure that the incidence of patients with CL is growing [5]. This is partially due to imposed environmental insults, which escalate human contact to the sandfly bite. Large-scale drought, desertification, climate change, population movement, and sporadic windstorms have imposed multiple serious ’downstream’ health effects in Iran’s southeastern provinces, more profoundly than before in Kerman, including Bam and Sistan/Baluchistan [48,49]. These factors were the primary drivers in creating hotspot areas for CL and led to extensive public health, social and economic costs. In Bam, drought influenced the ecosystem, thereby cascading negative impacts on food production, including crops and livestock, among many aspects. Decline water availability level is a prominent feature of prolonged droughts, contributed to further population motility, and abandoned their villages toward Kerman, Bam, and other neighboring cities within the province where the new emerging foci of ACL have occurred [17,19,21].

Another previous event that imposed marked changes as a side-benefit of anti-malaria activity and reduced sandflies numbers has previously been reported [26,50,51]. Following a long-lasting spraying withdrawal of domestic areas, an upsurge of leishmaniasis transmission was experienced. In a case-control study, the behavior of CL incidence following the termination of spraying in villages of Isfahan was studied. A 20-fold escalation of the new cases of ZCL after cessation of spraying with DDT was observed. The control communities did not show such variations [26].

In the past decades, a transition in the cultural behavior of agriculture has taken place. Nowadays, most agricultural practices are restricted to palm trees and orchards of oranges as sources of food and fruits. Growing date palms and orange trees are economically important professions and significant Bam investments. Unfortunately, a widespread Dubas bug (*Ommatissus* spp.) infestation imposed substantial damage to palm trees, resulting in the widespread use of systemic insecticides. Pyrethroids were used repeatedly against the nymphal stage and adult insects. Nowadays, date palms in Iraq, Iran, and Oman suffer significant infestations with this bug [52]. Over the last three decades, this insect species has become a significant pest of date palms in Bam due to its heavy infestations, severity, and subsequent economic damages. Repeated and systemic aerial application of pyrethroids, especially deltamethrin insecticide, had probably partial side-benefit in controlling the sandfly vector and reducing CL in the area. In addition to therapeutics such as meglumine antimoniate to treat the patients, partial side-benefits of single and triple doses of autoclaved *Leishmania major* (ALM) vaccine against ACL, strategic measures such as environmental management, improvement of housing conditions, large-scale setting up of deltamethrin-soaked shelters, and curtains, and different insecticides have selectively been used in the past decades [31,32]

Environmental disturbances, low substructure, health system disruption, meteorological variables, and human activities are tightly linked to CL incidence in vulnerable inhabitants, destroying wetlands, and altering the phlebotomine habitats, behaviors, and compositions. *Ph*. *sergenti* sand flies have broadly adapted and propagated in the suburban communities and within Bam, as confirmed by different investigators [53,54]. These complex events represent that *L*. *tropica* adapted its cycle well to human habitation in the area. Also, urbanization has substituted old-style crops with more productive ones, leading to variations in vector populations connected to changed patterns of dispersion and spatial distribution of sand flies in new ranges. Such proliferative conditions fostered the rate of sandfly density, and enhanced human contact with the principal vector could potentially surge cases and perhaps drop future control programs’ effectiveness [55,56]. Changes in climatic factors are likely to lengthen the transmission seasons of important VBD and alter their geographical range, dispersal, and abundance [57].

Consistent with many other VBDs, leishmaniasis is highly susceptible to ecological changes motivated by anthropogenic and natural actions since its emergence depends on the environmental balance between diverse parasites, host, and vector species in complex transmission environments [58]. Natural disasters, especially earthquakes, can enhance and foster a breeding ground for the spread and outbreaks of VBD like CL and VL [59,60].

Cross-border movement and tourism from neighboring endemic countries for CL are other major challenges for a comprehensive control program. In addition, CL is more closely related to rural seasonal laborers, returnees, and Afghan migrants in this area. They have played a crucial role in disseminating the disease and introducing new emerging epidemics in small towns and even rural communities within Kerman province [8,17–21].

A specific long-term governmental budget was allocated to control ACL through the MOHME following the devastating earthquake in Bam. The budget allocation was sustained for seven years (2003–2008). The earthquake in Bam was the primary impetus to develop the first national guideline for surveillance and management of CL in Bam [61]. Following this crisis, many extensive efforts were devoted to training health personnel and staff, face-to-face health education of the community households, active and passive case detection surveillance, modification of the physical environment, and solid waste management. These interventions have been more or less sustained up to the present day to reduce the incidence rate and control the disease. The health center in charge of CL control and its long-term activities has become a control model for the Eastern Mediterranean Region and has held workshops and trained staff from different endemic countries.

Furthermore, the schools’ employees were granted financial incentives to improve primary and secondary schools’ health conditions (1993–1998). An extensive systematic dog population management (2003–2008) was conducted because of rabies, CL, and other helminths and pathogens. The action had implications for public health, wildlife, and animal welfare. Sheltering was the most common approach to free-roaming dog population control [39].

All age groups were affected in Bam county, but most cases happened in those ≥ 21 years old (p < 0.001). The higher infection rate in this age group, particularly in male individuals (p < 0.001), is due to the arrival and participation of male health workforces from different counties in Bam for service offering and occupational purposes. Most of these health providers were male and remained in the area for one year after the earthquake. Therefore, the infected male to female population was significantly (p < 0.001) higher after the earthquake, unlike the previous events. Also, different risk determinants have been associated with a higher incidence in male subjects. Male patients, especially ≥ 21 years of age, were more involved in providing municipal services and thus became more infected than females. Furthermore, male patients do not often follow the option of therapy with conventional drugs, mainly because of poor compliance with therapy [29]. On the other hand, females are more disciplined and cover their bodies entirely, are limited to their houses, and are exposed less frequently to the source of infection.

The present results also demonstrated that the skin lesions were most commonly found on the hands and face. These findings are consistent with previous outcomes that showed that these anatomical locations were the general site of CL involvement [8,18,62]. *L*. *tropica*, the causal parasite of ACL, was the only causative agent identified after the earthquake in Bam [63–65]. However, a report indicates that *L*. *major*, causing ZCL was sporadically reported from the rural communities of Bam county [66].

This observation highlights the enormous improvements made in CL surveillance during the last two decades in this area. Nevertheless, sustained funding and more trained task forces are essential to strengthen surveillance further and implement robust control strategies. Hopefully, the current WHO NTDs roadmap 2021–2030 suggests more robust indicators and targets to monitor the CL control and elimination plan [67].

To our knowledge, this historical observation represents a unique and long-lasting study to assess natural and man-made events on excessive scales for patients with ACL worldwide. First, the solid central point of this study is the CL control clinic with a vigorous registry and surveillance system and adequate resources and technical infrastructures to carry out environmental interventions and manage the cases through a group of well-trained staff, physicians, and equipped diagnostic services, funded by Bam and Kerman health networks and universities. Second, the first and corresponding authors and some co-authors were directly involved and carefully monitored the work because of their field of interest and health authorities in Bam and Kerman universities and health systems.

Nevertheless, the longitudinal investigation had several limits. First, even though CL is associated with the health surveillance system, a limitation was the absence of active finding approaches systematically to follow-up and examine the patients by the house–to–house appointments. Active case detection policies could help assess the genuine burden of CL in the place. Second, before 1993, all data were routinely gathered by the health networks and clinics in Bam based on the passive case finding strategy. Aflatoonian, the first author who carried out his thesis under the supervision of Professor Nadim in 1988, gathered previous data [26].

In conclusion, following significant man-made and natural environmental changes such as earthquakes, many determinants could activate CL outbreaks in a long-standing endemic focus and induce emerging foci in new areas. Multiple complex factors, human activities, and natural environmental events, including cessation of anti-malarial spraying activities, fast and unplanned urbanization, disasters, and widespread migration, were among the leading causes that synergistically induced the emergence/re-emergence of ACL repeated epidemic waves in this part of the country. Since human beings are the principal reservoir host for ACL, timely detection and effective therapy of cases are the primary control measures to reduce the disease’s burden. However, the most crucial evidence gaps remain, and new tools are crucial before CL can ultimately be controlled. Moreover, setting up early warning systems for NTDs, particularly CL, can help predict CL outbreaks. Therefore, essential readiness and speedy response mechanisms must be in place in highly vulnerable localities within endemic countries.

## Supporting information

S1 STROBE Checklist STROBE Statement—Checklist of items that should be included in reports of observational studies. (DOCX)
